# Effects of Haima Duobian Pill in a Rat Model of Kidney Yang Deficiency Syndrome

**DOI:** 10.1155/2021/6696234

**Published:** 2021-01-05

**Authors:** Yijia Zeng, Tingna Li, Xiaorui Zhang, Yuanyuan Ren, Qinwan Huang, Jin Wang, Li Yuan, Funeng Geng

**Affiliations:** ^1^Chengdu University of Traditional Chinese Medicine, Chengdu, Sichuan 611137, China; ^2^Sichuan Engineering Research Center for Medicinal Animals, Chengdu, Sichuan 610031, China

## Abstract

**Objective:**

Modern research shows that Haima Duobian pill (HDP) can relieve the kidney yang deficiency syndrome (KYDS), but the mechanism is still unclear. The aim of this work was to study the effects of HDP in a rat model of KYDS.

**Materials and Methods:**

The network pharmacology methods were used to predict the therapeutic effects of Haima Duobian pill. Adenine was used to establish the rat model of kidney yang deficiency syndrome. The general physical signs of rats were observed after different doses of Haima Duobian pill (HDP) were given. Serum cyclic adenosine monophosphate (cAMP), cyclic guanosine monophosphate (cGMP), luteinizing hormone (LH), follicle-stimulating hormone (FSH), testosterone (T), estradiol (E2), and gonadotropin-releasing hormone (GnRH) levels were determined using enzyme-linked immunosorbent assay (ELISA) kits. Then, the histopathologic changes and sperm activity were detected.

**Results:**

HDP could improve the general signs of kidney yang deficiency syndrome rats. After the rats were treated with HDP, the expression of cGMP and E2 was significantly inhibited and the expression of cAMP and T was significantly increased. The pathological damage of testis, epididymis, and seminal vesicle was alleviated, and the sperm activity was improved.

**Conclusion:**

For adenine-induced kidney yang deficiency syndrome in rats, HDP had a significant therapeutic effect.

## 1. Introduction

Kidney yang deficiency syndrome (KYDS), a diagnostic pattern in Chinese medicine, has clinical features similar to those of the glucocorticoid withdrawal syndrome [1]. It is a metabolic disease caused by a neuroendocrine disorder [2]. The main clinical manifestations of the male patients with KYDS are infertility in the childbearing age, decreased sexual desire or frigidity, less semen, low sperm activity, waist pain, and more nocturia. Severe cases may even lead to spermatogenic dysfunction, resulting in infertility and birth defects affecting at least 5 million families [3]. Modern research has found that the dysfunction of hypothalamic-pituitary-gonadal axis (HPG axis) is the basis of KYDS, and the HPG axis consists of three levels: hypothalamus, pituitary, and gonads (testes in the male and ovaries in the female) [4].

Haima Duobian pill (HDP) is a classic traditional Chinese medicine (TCM) formula for the treatment of KYDS and has been widely used to recuperate KYDS clinically for hundreds of years in China. There are few studies on the mechanism of HDP action. Based on the HPG axis, this study will explore the effect of HDP on spermatogenesis in rats with KYDS.

## 2. Materials and Methods

### 2.1. Screening of Active Compounds of Haima Duobian Pill

The Traditional Chinese Medicine Systems Pharmacology (TCMSP) database was used to screen the bioactive compounds. The ADME parameter-based virtual screening of the compounds was utilized to further identify compounds using an oral bioavailability (OB) threshold OB ≥30% and a drug-likeness (DL) threshold DL ≥0.18.

### 2.2. Prediction of Chemical Component Targets

TCMSP was used to search for potential targets associated with active components.

### 2.3. Screening of the Targets Related to the Dysfunction of Hypothalamic-Pituitary-Gonadal Axis

With “dysfunction of hypothalamic-pituitary-gonadal axis” as the key word, the research team searched OMIM (https://https://omim.org/) and GeneCards (http://www.genecards.org/) databases and got 1122 disease-related targets.

### 2.4. The Target Prediction of Drug Action

The effective targets of Haima Duobian pill and the related targets of the dysfunction of hypothalamic-pituitary-gonadal axis were analyzed with R programming language. A total of 46 intersection targets were obtained. The online STRING database (https://string-db.org/) was used to visualize the information.

### 2.5. Experiments for Verification

#### 2.5.1. Quality Control of Haima Duobian Pill

**Figure 1:**
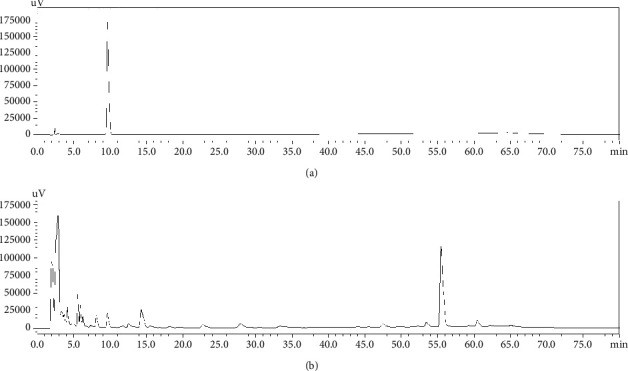
The HPLC chromatograms of (a) Schisandrol A and (b) HDP.

The quality control of Haima Duobian pill was analyzed using high-performance liquid chromatography (HPLC) with photodiode array (PDA) detection (Shimadzu, Japan). The powder of Haima Duobian pill was extracted with water saturated ethyl acetate. The extract was evaporated to dryness. The residue was dissolved in methanol. The solution was filtered, and then 10 *μ*L was injected into an HPLC system for assay. The separation was performed using a reverse-phase column (Eclipse Plus C18, 5 *μ*m, 4.6 × 250 mm ID, Agilent Technologies, USA), with the column temperature at 30°C. The elution flow rate was 1.0 mL/min with a mobile phase gradient of *A* − *B* (*A*: 0.02% H_3_PO_4_; *B*: C_2_H_3_N), which was varied as follows: 0–30 min, 51% *A*; 30–60 min, 51%–80% *A*; 60–70 min, 80%–51% *A*; 70–80 min, 51% *A*. Schisandrol A was detected at UV 210 nm. The HPLC chromatogram of Haima Duobian pill is shown in Figure 1.

#### 2.5.2. Experimental Animals and Groups

SD male rats, weighing 200–300 g, were provided by Chengdu Dashuo Experimental Animal Co., Ltd., production permit: SCXK (Chuan) 2015-030. The rats were fed adaptively for 3 days, during which they could drink freely. Then, the rats were divided into 6 groups (12 rats in each group): normal group (NG), model group (MG), high dose group (HDG), middle dose group (MDG), low dose group (LDG), and positive group (Wuzi Yanzong pill group, PG). The experiment was completed in the Pharmacology Laboratory of Traditional Chinese Medicine of Chengdu University of Traditional Chinese Medicine. The laboratory is the Research Laboratory of Traditional Chinese Medicine of the State Administration of Traditional Chinese Medicine (Level III), Certificate Registration No. TCM-2009-315.

#### 2.5.3. Experimental Drugs

Haima Duobian pill (batch No. 190410, Shenyang Qinggong Pharmaceuticals Group Co., Ltd.) was used. Haima Duobian pill is composed of Haima (*Hippocampus*), Gejie (*Gekko gecko*), Jiucaizi (*Allii Tuberosi Semen*), Suoyang (*Cynomorium songaricum Rupr*.), Lurong (*Cornu cervi pantotrichum*), Buguzhi (*Psoralea corylifolia Linn*.), Xiaohuixiang (*Foeniculi Fructus*), Tusizi (*Cuscutae Semen*), Shayuanzi (*Astragali Cpmplanatisemen*), Shanzhuyu (*Cornus Officinalis Sieb. et Zucc*.), Baizhu (*Atractylodes macrocephala Koidz*.), Duzhong (*Eucommiae Cortex*), Hongshen (*Ginseng Radix et Rhizoma Rubra*), Mudingxiang (Fruit of *Caryophylli Flos*), Niuxi (*Achyranthis Bidentatae Radix*), Fuling (*Poria cocos (Schw.) Wolf.*), Shanyao (*Rhizoma Dioscoreae*), Huangqi (*Hedysarum multijugum Maxim*.), Danggui (*Angelicae Sinensis Radix*), Longgu, Gancao (*Licorice*), Rougui (*Cinnamomi Cortex*), Quenao (*Brain marrow of Passer montanus (Linnaeus)*), Wuweizi (*Schisandrae Sphenantherae Fructus*), Gouqizi (*Lycii Fructus*), Goubian (*Dog whip*), Lvbian (*Donkey whip*), Niubian (*Bullwhip*), Diaobian (*Mink whip*), Shudihuang (*Rehmanniae Radix Praeparata*), Fuzi (*Aconiti Lateralis Radix Praeparata*), Roucongrong (*Cistanches Herba*), Bajitian (*Morinda officinalis Radix*), Yinyanghuo (*Epimedii Herba*). Wuzi Yanzong pill (batch No. 18035165, Tongrentang Pharmaceutical Factory of Beijing Tongrentang Co., Ltd.), Schisandrol A (Lot: MUST-19031905, Chengdu Must Bio-Technology Co., Ltd.), Adenine (batch No. T14N9Y74844, Shanghai YuanYe Biotechnology Co., Ltd.), Rat LH (Luteinizing Hormone) ELISA Kit (Lot: 3XNQ4LLQH7), Rat FSH (follicle-stimulating hormone) ELISA Kit (Lot: UT7DCSJV5Y), cAMP (cyclic adenosine monophosphate) ELISA Kit (Lot: UA4SG5RCJW), GnRH (gonadotropin-releasing hormone) ELISA Kit (Lot: 1TP7S3C8HH), cGMP (cyclic GMP) ELISA Kit (Lot: K6LFPAPG7N), T (Testosterone) ELISA Kit (Lot: K4P1SC1WZS), and Rat/Porcine E2 (Estradiol) ELISA Kit (Lot: CL8NR4F5FH). All of the above kits were purchased from Wuhan Yilairuite Biotechnology Co., Ltd.

#### 2.5.4. KYDS Rat Model and Treatment

The rat model of kidney yang deficiency syndrome was established by intragastric administration of adenine for 21 days, with a daily dose of 200 mg/kg. From the 22nd day, except for the normal group and the model group, all groups were administered by gavage, among which the intake for the high dose group was 1.6 g/kg, the middle dose group was 0.8 g/kg, the low dose group was 0.4 g/kg, and the positive group was 2.4 g/kg. Rats in each group were administered by gavage once a day for 21 days.

#### 2.5.5. Observation of Signs and Symptoms

During the experiment, the general physical signs of rats were observed every other week, including body weight, anal temperature, food intake, water intake, depilation, urine output, and defecation.

*(1) Organ Index Test*. After intraperitoneal injections of sodium pentobarbital, the kidney, testis, epididymis, and seminal vesicle gland of male rats were extracted and weighed, and the organ index of each organ or tissue was calculated by the following formula: organ index = organ weight/body weight × 100%.

*(2) Measurement of Serum Cytokines*. After intraperitoneal injections of sodium pentobarbital, 5 ml of abdominal aortic blood was taken from each rat (rats were able to drink freely 12 hours before taking blood). After 2 hours, the blood samples were centrifuged at 3000 *r*/min for 10 min, and the serum was taken. The contents of cAMP, cGMP, GnRH, LH, FSH, T, and E2 in the serum were determined by ELISA Kit.

*(3) Sperm Activity Test*. After intraperitoneal injections of sodium pentobarbital, the epididymis of each rat was taken. It was put into 8 ml normal saline and cut to pieces to make its distribution uniform. The suspension was put in a 37°C water bath for 10 min and filtered, and normal saline was added to 8 ml and prepared as sperm suspension. One drop of sperm suspension was taken and observed under microscope. According to the WHO manual, sperm motility was divided into four grades: *A* (fast forward movement), *B* (slow forward movement), *C* (nonforward movement), and *D* (no movement). The ratio of *A* + *B* grade sperm was calculated [5, 6].

*(4) Histopathological Observation*. The organs were fixed and observed under microscope after HE staining.

*(5) Statistical Analysis*. SPSS 19.0 was used to analyze the data. Gaussian distribution of each data was evaluated using the Shapiro–Wilk normality test. Differences among groups were analyzed using one-way analysis of variance (ANOVA). Normally distributed measurement data were expressed as the mean ± standard deviation (SD). *P* < 0.05 showed that the difference was statistically significant.

## 3. Results

### 3.1. The Prediction of the Targets of Haima Duobian Pill in the Treatment of KYDS and the Analysis of Interaction between the Targets

**Figure 2:**
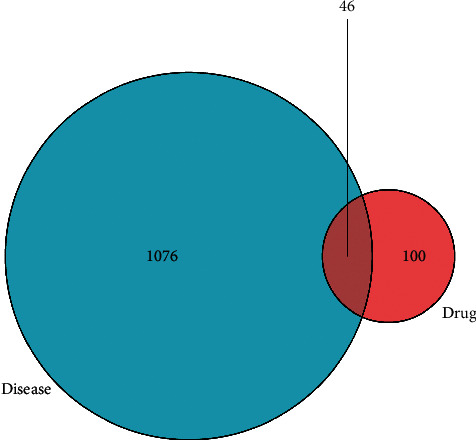
Venn diagram of the intersection targets. The intersection part represented the common targets.

**Figure 3:**
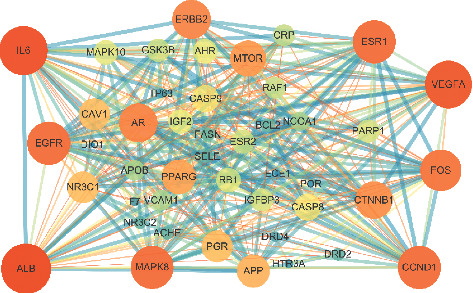
PPI network of the 45 intersection targets. The larger the degree value of the node was, the larger the node size was, and the brighter the node color was. The larger the combined score was, the larger the edge size was, and the darker the color was.

A total of 1122 disease-related targets, 146 drug-related targets, and 46 intersection targets were identified. The result is shown in Figure 2. The intersection targets were imported into STRING with the gene type selected as *Homo sapiens*. Setting the medium confidence to 0.400 and hiding the disconnected nodes in the network, we could obtain the protein-protein interaction information (Figure 3). The network comprised 46 nodes and 372 edges. The average node degree was 16.2, and the local clustering coefficient was 0.725. Network pharmacological analysis showed that Haima Duobian pill could act on multiple targets related to the dysfunction of hypothalamic-pituitary-gonadal axis, and there was a multilevel interaction between these targets. Therefore, Haima Duobian pill may regulate the dysfunction of hypothalamic-pituitary-gonadal axis through multiple targets and pathways to treat KYDS.

### 3.2. General Signs

Before modeling, the rats in each group had normal activity, smooth fur, sensitive response, and normal diet. After 21 days of modeling, each model rat showed the symptoms of KYDS, such as emaciation, crouching back, chilly limbs, loose and yellow body hair, lack of luster, mental malaise, slow response, more drinking water, less eating, and more urine. After 21 days of administration, the symptoms of KYDS in high dose, middle dose, low dose, and positive groups were improved, and the symptoms in model group were also improved, but the improvement was not obvious. The changes of physical signs in normal group were not obvious.

### 3.3. Detection of Weight Growth Rate and Body Temperature

**Figure 4:**
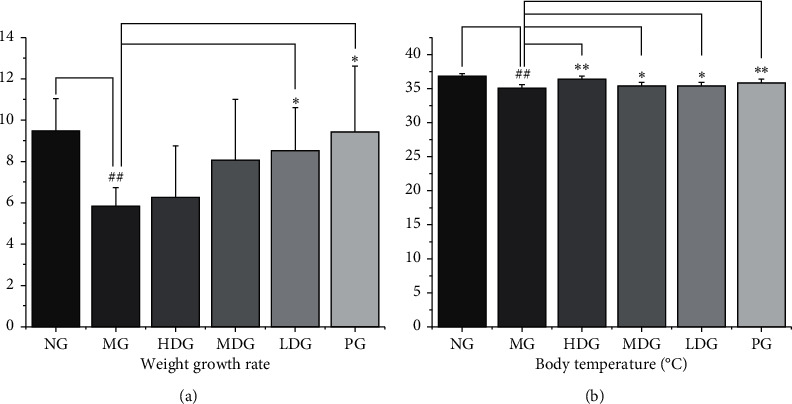
Symptom recording during the experiment. No significant difference was reported in body weight and body temperature among the six groups before the experiment. (a) Changes of body weight growth rate in rats treated with or without HDP, df = 68, *F* = 5.005. Compared with the normal group, the weight growth rate of the model group significantly reduced. Compared with the model group, the weight growth rate of the low dose group and the positive group increased significantly. (b) Changes of body temperature in rats treated with or without HDP, df = 68, *F* = 25.609. Compared with the normal group, the body temperature of the model group was significantly lower. Compared with the model group, the body temperature of high dose, middle dose, low dose, and positive groups was significantly higher. The data of each group were expressed as mean ± standard deviation (SD). ^##^*P* < 0.01 and ^#^*P* < 0.05 versus normal group; ^*∗∗*^*P* < 0.01 and ^*∗*^*P* < 0.05 versus model group. NG: normal group, MG: model group, HDG: high dose group, MDG: middle dose group, LDG: low dose group, and PG: positive group.

First, we determined the differences in weight growth rate among the groups. The results are shown in Figure 4. Compared with the blank group, the weight growth rate of the model group was significantly reduced (*P* < 0.01). Compared with the model group, the weight growth rate of the low dose group and the positive group increased significantly (*P* < 0.05). After that, we analyzed the body temperature of each group of rats, as shown in Figure 4. Compared with the blank group, the body temperature of the model group was significantly lower (*P* < 0.01). Compared with the model group, the body temperature of high dose, middle dose, low dose, and positive groups was significantly higher (*P* < 0.01 or *P* < 0.05).

### 3.4. Effect on Organ Index

**Figure 5:**
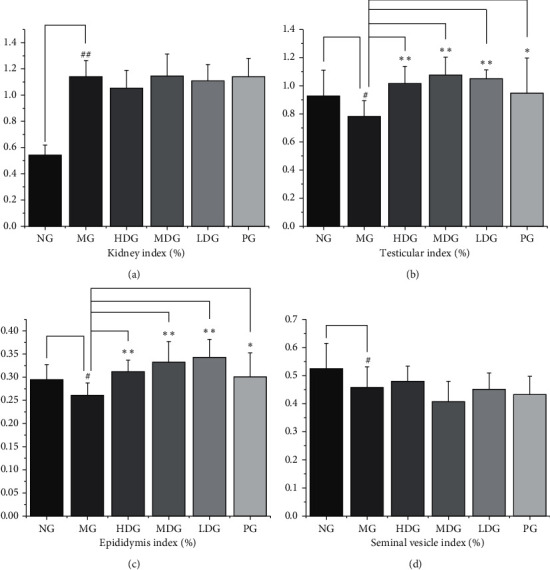
*Organ index of kidney, testis, epididymis, and seminal vesicle gland.* (a) Kidney, df = 66, *F* = 38.719. Compared with the normal group, the kidney index of the model group was significantly higher. (b) Testis, df = 66, *F* = 4.945. Compared with the normal group, the testicular index of the model group was lower. Compared with the model group, the testicular index of the high dose group, the middle dose group, the low dose group, and the positive group was higher. (c) Epididymis, df = 66, *F* = 6.252. Compared with the normal group, the epididymis index of the model group was lower. Compared with the model group, the epididymis index of the high dose group, the middle dose group, the low dose group, and the positive group was higher. (d) Seminal vesicle gland, df = 66, *F* = 3.924. Compared with the normal group, the seminal vesicle index of the model group was lower. The data of each group were expressed as mean ± standard deviation (SD). ^##^*P* < 0.01 and ^#^*P* < 0.05 versus normal group; ^*∗∗*^*P* < 0.01 and ^*∗*^*P* < 0.05 versus model group. NG: normal group, MG: model group, HDG: high dose group, MDG: middle dose group, LDG: low dose group, PG: positive group.

Compared with the normal group, the kidney index of the model group was significantly higher (*P* < 0.01). It may be due to adenine deposition. Compared with the model group, the renal index of the high dose group of HDP was lower, but the difference was not statistically significant (*P* > 0.05). Compared with the normal group, the testis index of the model group was lower, and the difference was statistically significant (*P* < 0.05). Compared with the model group, the testis index of the high dose group, the middle dose group, and the low dose group of HDP was higher, and the difference was statistically significant (*P* < 0.01). The testis index of the positive group was higher than that of the model group (*P* < 0.05). Compared with the normal group, the epididymis index of the model group was lower, and the difference was statistically significant (*P* < 0.05). Compared with the model group, the epididymis index of the high dose group, the middle dose group, and the low dose group of HDP was higher, and the difference was statistically significant (*P* < 0.01). The epididymis index of the positive group was also higher than that of the model group, and the difference was statistically significant (*P* < 0.05). Compared with the normal group, the seminal vesicle index of the model group was lower, and the difference was statistically significant (*P* < 0.05). Compared with the model group, the seminal vesicle index of the high dose group of HDP was higher, but the difference was not statistically significant (*P* > 0.05). These results indicate that HDP has the tendency to improve the atrophy of gonadal axis target organs. The results are shown in Figure 5.

### 3.5. Effect on cAMP, cGMP, and cAMP/cGMP in Serum

**Figure 6:**
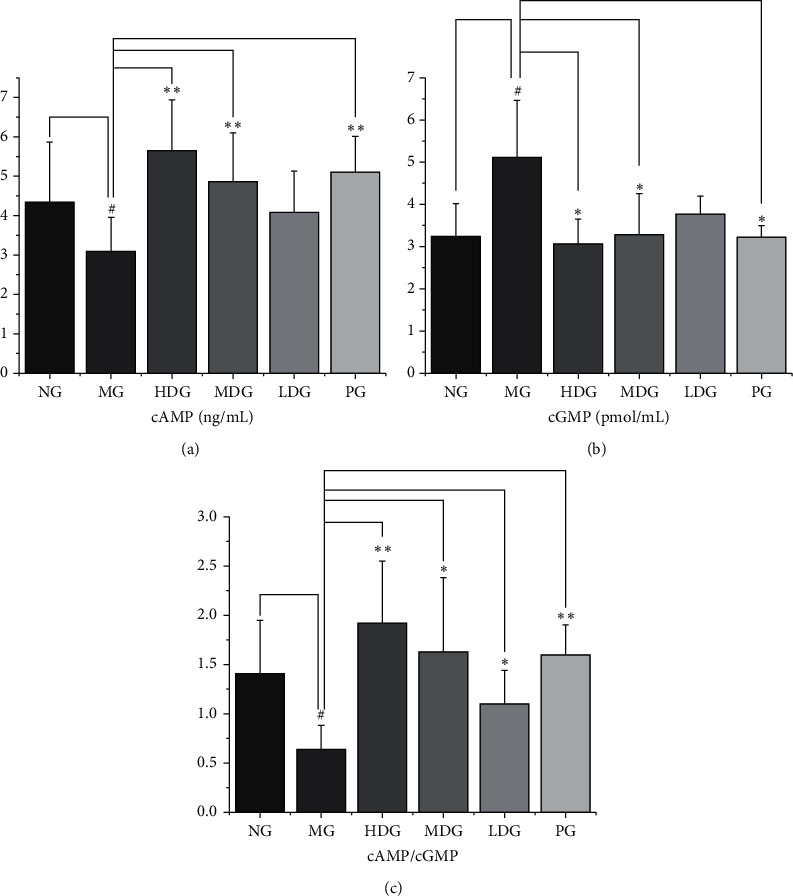
*The levels of cAMP, cGMP, and cAMP/cGMP in serum.* (a) cAMP, df = 59, *F* = 5.88. Compared with the normal group, the serum cAMP of the model group was lower. Compared with the model group, the serum cAMP of the high dose, middle dose, and positive group was higher. (b) cGMP, df = 59, *F* = 8.951. Compared with the normal group, the cGMP content of the model group was higher. Compared with the model group, the cGMP content of the high dose group, the middle dose group, and the positive group was lower (c) cAMP/cGMP, df = 59, *F* = 8.067. Compared with the normal group, the cAMP/cGMP of the model group was lower. Compared with the model group, the serum cAMP/cGMP of the high dose group, the middle dose group, the low dose group, and the positive group was higher. ELISA was performed according to the manufacturer's protocol. The data of each group were expressed as mean ± standard deviation (SD). ^##^*P* < 0.01 and ^#^*P* < 0.05 versus normal group; ^*∗∗*^*P* < 0.01 and ^*∗*^*P* < 0.05 versus model group. NG: normal group, MG: model group, HDG: high dose group, MDG: middle dose group, LDG: low dose group, PG: positive group.

Compared with the normal group, the serum cAMP of the model group was lower, and the difference was statistically significant (*P* < 0.05). Compared with the model group, the serum cAMP of the high dose and middle dose groups of HDP and the positive group was higher, and the difference was statistically significant (*P* < 0.01). Compared with the normal group, the cGMP content in the serum of the model group was higher, and the difference was statistically significant (*P* < 0.05). Compared with the model group, the cGMP content in the serum of the high dose group, the middle dose group, and the positive group was lower, and the difference was statistically significant (*P* < 0.05). Compared with the normal group, the cAMP/cGMP in the serum of the model group was lower, and the difference was statistically significant (*P* < 0.05). Compared with the model group, the serum cAMP/cGMP in the high dose group, the middle dose group, and the low dose group of HDP was higher, with statistical significance (*P* < 0.01 or *P* < 0.05). The serum cAMP/cGMP in the positive group was higher than that in the model group, with statistical significance (*P* < 0.01). It can be concluded that HDP can regulate the levels of cAMP and cGMP in the serum of rats with KYDS. The results are shown in Figure 6.

### 3.6. Effects on the Levels of LH, FSH, T, E2, and GnRH in Serum

**Figure 7:**
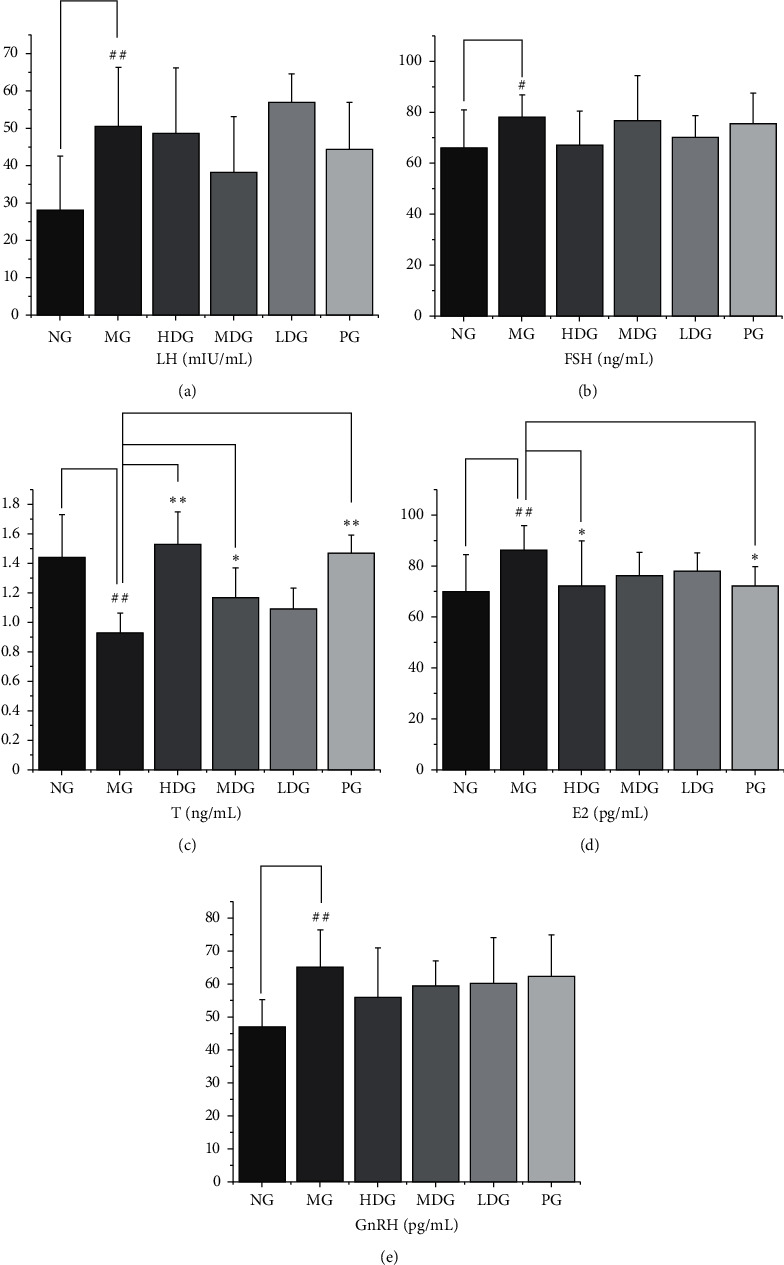
*The levels of LH, FSH, T, E2, and GnRH in serum.* (a) LH, df = 56, *F* = 4.716. Compared with the normal group, the serum LH level of the model group was higher. (b) FSH, df = 59, *F* = 1.597. Compared with the normal group, the level of FSH in the serum of the model group was higher. Compared with the model group, there was no significant difference in serum FSH level of the other administration groups. (c) T, df = 55, *F* = 14.23. Compared with the normal group, the level of T of the model group was lower. Compared with the model group, the level of T of the high dose group, the middle dose group, and the positive group was higher. (d) E2, df = 54, *F* = 2.395. Compared with the normal group, the serum E2 content of the model group was higher. Compared with the model group, the serum E2 content of the high dose group and positive group was lower. (e) GnRH, df = 51, *F* = 2.848. Compared with the normal group, the level of GnRH of the model group was higher. ELISA was performed according to the manufacturer's protocol. The data of each group were expressed as mean ± standard deviation (SD). ^##^*P* < 0.01 and ^#^*P* < 0.05 versus normal group; ^*∗∗*^*P* < 0.01 and ^*∗*^*P* < 0.05 versus model group. NG: normal group, MG: model group, HDG: high dose group, MDG: middle dose group, LDG: low dose group, PG: positive group.

Compared with the normal group, the serum LH level of the model group was higher, and the difference was statistically significant (*P* < 0.01); compared with the model group, the difference of serum LH level of the other groups had no statistical significance (*P* > 0.05). Compared with the normal group, the level of FSH in the serum of the model group was higher, and the difference was statistically significant (*P* < 0.05). Compared with the model group, there was no significant difference in serum FSH level of the other administration groups (*P* > 0.05). Compared with the normal group, the content of T in the serum of the model group was lower, and the difference was statistically significant (*P* < 0.01). Compared with the model group, the content of T in the serum of the high dose group, the middle dose group, and the positive group was higher, and the difference was statistically significant (*P* < 0.01 or *P* < 0.05). Compared with the normal group, the serum E2 content of the model group was higher, and the difference was statistically significant (*P* < 0.01). Compared with the model group, the serum E2 content of the high dose group and positive group was lower, and the difference was statistically significant (*P* < 0.05). Compared with the normal group, the content of GnRH in the serum of the model group was higher, and the difference was statistically significant (*P* < 0.01). Compared with the model group, there was no significant difference in the serum GnRH level of administration group (*P* > 0.05). These results indicate that HDP can regulate the secretion of cytokines, increase the level of T, and improve the clinical symptoms of KYDS. The results are shown in Figure 7.

### 3.7. Sperm Activity Test

**Figure 8:**
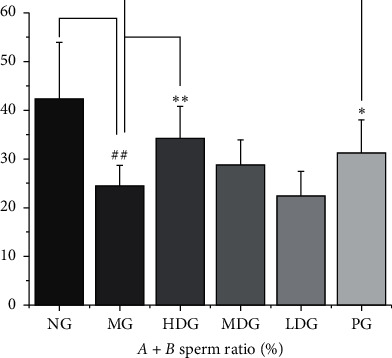
*Sperm activity test,* df = 60, *F* = 10.23. Compared with the normal group, the *A* + *B* grade sperm ratio of the model group was significantly lower. Compared with the model group, the *A* + *B* grade sperm ratio of the high dose group and positive group was significantly higher. The data of each group were expressed as mean ± standard deviation (SD). ^##^*P* < 0.01 and ^#^*P* < 0.05 versus normal group; ^*∗∗*^*P* < 0.01 and ^*∗*^*P* < 0.05 versus model group. NG: normal group, MG: model group, HDG: high dose group, MDG: middle dose group, LDG: low dose group, PG: positive group.

Compared with the normal group, the ratio of *A* + *B* grade spermatozoa in the model group was significantly lower, and the difference was statistically significant (*P* < 0.01). Compared with the model group, the ratio of *A* + *B* grade sperm in the high dose group and positive group increased significantly (*P* < 0.01, *P* < 0.05). The results are shown in Figure 8.

### 3.8. Histopathological Observation

#### 3.8.1. Pathological Observation of Kidney

**Figure 9:**
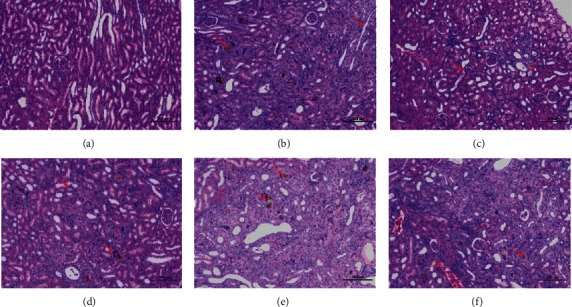
*Pathological observation of kidney.* Kidney tissues obtained at the end of the experiment were stained with hematoxylin and eosin (H&E, 100x magnification): (a) NG; (b) MG; (c) HDG; (d) MDG; (e) LDG; (f) PG. NG: normal group, MG: model group, HDG: high dose group, MDG: middle dose group, LDG: low dose group, PG: positive group. Scale bar = 200 *μ*m.

The boundary between renal cortex and medulla was clear in normal group. The number of renal tubules was abundant, and the epithelial cells were normal. The renal volume of model group and other administration groups increased. A large number of adenine crystals were seen, some of which were encapsulated by macrophages and multinucleated macrophages, forming granuloma. More renal tubules were atrophied, the lumen became narrower, and the volume of epithelial cells decreased. The basement membrane of renal tubules was thickened. There were more connective tissue hyperplasia and inflammatory cell infiltration in renal tubules. The results are shown in Figure 9.

#### 3.8.2. Pathological Observation of Testis

**Figure 10:**
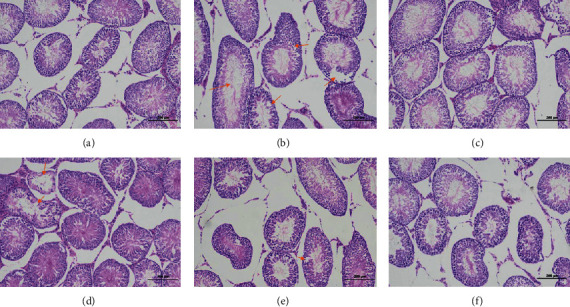
Pathological observation of testis. Testis tissues obtained at the end of the experiment were stained with hematoxylin and eosin (H&E, 100x magnification): (a) NG; (b) MG; (c) HDG; (d) MDG; (e) LDG; (f) PG. NG: normal group, MG: model group, HDG: high dose group, MDG: middle dose group, LDG: low dose group, PG: positive group. Scale bar = 200 *μ*m.

In the normal group, the shape of seminiferous tubules was regular. The spermatogenic cells and Sertoli cells in tubules were closely arranged and abundant, and long fusiform spermatozoa could be seen. No other obvious abnormality was found in the tissues. In the model group, the arrangement of spermatids in the seminiferous tubules was disordered. The connective tissue between the seminiferous tubules was increased. The number of interstitial cells was significantly reduced. The number of spermatozoa was greatly reduced, and the seminiferous cells were exfoliated in some tubules. In the high dose group, the shape of seminiferous tubules was regular, and the gap was widened. The spermatogenic cells were arranged closely and abundant. Long fusiform spermatozoa could be seen, and no other obvious abnormality was found in the tissues. The shape of seminiferous tubules in the middle dose group was regular, and the gap was widened. A small number of seminiferous tubules had a large number of decreased spermatocytes, and no other obvious abnormality was found in the tissues. In the low dose group, the shape of seminiferous tubules was regular, and the gap was widened. The spermatogenic cells were closely arranged, and long fusiform spermatozoa could be seen. The number of spermatozoa in some seminiferous tubules was significantly reduced, and no other obvious abnormality was found in the tissues. In the positive group, the shape of seminiferous tubules was regular, and the gap was widened. The spermatogenic cells were arranged closely and abundant, and there were long fusiform spermatozoa in the tubules. No other obvious abnormality was found in the tissues. The results are shown in Figure 10.

#### 3.8.3. Pathological Observation of Epididymis

**Figure 11:**
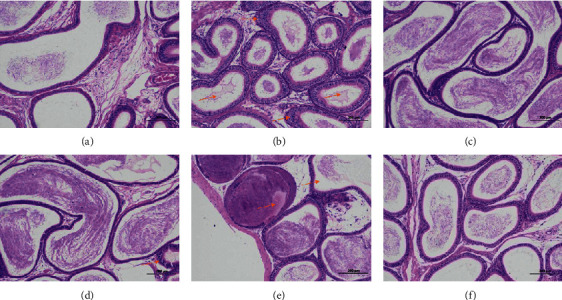
Pathological observation of epididymis. Epididymis tissues obtained at the end of the experiment were stained with hematoxylin and eosin (H&E, 100x magnification): (a) NG; (b) MG; (c) HDG; (d) MDG; (e) LDG; (f) PG. NG: normal group, MG: model group, HDG: high dose group, MDG: middle dose group, LDG: low dose group, PG: positive group. Scale bar = 200 *μ*m.

In the normal group, the epithelial cells of epididymis were normal in morphology, with clear boundary and no other obvious abnormality. In the model group, the epididymal epithelial cells were necrosis, and the nuclei were pyknosis, hyperchromatism, or fragmentation. The lumen was filled with sperm and eosinophilic protein fluid. Interstitial connective tissue was loose, and a small number of inflammatory cells were infiltrated. In the high dose group, the epithelial cells of epididymis were normal in morphology, with clear boundary and no other obvious abnormality. In the middle dose group, some epididymal epithelial cells were swollen. The cytoplasm was loose and light stained. No other obvious abnormality was found. In the low dose group, the epididymal epithelial cells swelled and the number of sperm in some lumens decreased significantly. In the positive group, the morphology of epididymal epithelial cells was normal, the boundary was clear, and the interstitial connective tissue was loose. There was no other obvious abnormality. The results are shown in Figure 11.

#### 3.8.4. Pathological Observation of Seminal Vesicle Gland

**Figure 12:**
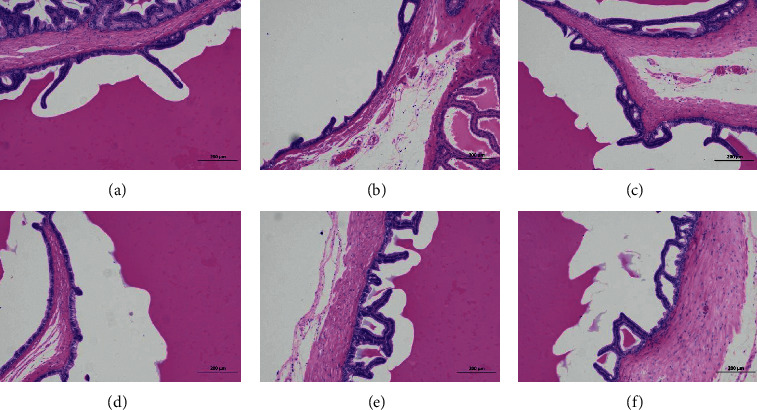
Pathological observation of seminal vesicle gland. Seminal vesicle gland tissues obtained at the end of the experiment were stained with hematoxylin and eosin (H&E, 100x magnification): (a) NG; (b) MG; (c) HDG; (d) MDG; (e) LDG; (f) PG. NG: normal group, MG: model group, HDG: high dose group, MDG: middle dose group, LDG: low dose group, PG: positive group. Scale bar = 200 *μ*m.

In the normal group, the structure of each layer of seminal vesicle was clear. The mucosal layer and muscular layer were closely arranged, and a series of folds could be seen in the mucosal layer. The epithelium of the mucosal layer was a single columnar epithelium, and the epithelial cells were closely arranged with normal morphology. No other obvious abnormality was found in the tissue. The seminal vesicles of other groups were dilated, and no other obvious tissue abnormality was found. The results are shown in Figure 12.

## 4. Discussion

Kidney yang deficiency syndrome (KYDS), a metabolic disease caused by a neuroendocrine disorder, was recorded first in an early systematic and theoretical monograph existing in China, “Neijing” [7]. The theory of TCM proposes that the physiological function of the body would be at a low level in KYD syndrome. Studies have found that the pathogenesis mechanism of KYDS is mainly in the multilevel dysfunction of the hypothalamic-pituitary-target gland axis (adrenal, thyroid, and gonad). It is characterized by qi deficiency, cold limbs, decreased mobility, slow response, decreased appetite, cowered, polyuria, diarrhea, and sparse hair, etc. [8–10]. In this study, we observed that KYDS rat model showed lower body weight growth rate and body temperature than normal rats, and the model group rats generally had the symptoms of emaciation, curling and arching back, chilly limbs, yellow and loose body hair, mental weakness, slow response, increased drinking water, reduced diet, and polyuria. These results are similar to those reported in the literature.

Reproduction is controlled by the hypothalamic-pituitary-gonadal (HPG) axis. Gonadotropin-releasing hormone (GnRH) neurons can produce GnRH and stimulate the biosynthesis and secretion of follicle-stimulating hormone (FSH) and luteinizing hormone (LH). GnRH is the main hypothalamic regulator of the release of gonadotropins. FSH and LH regulate the function of testis through their receptors in Sertoli and Leydig cells, respectively [11]. LH, FSH, and LH-stimulated high intratesticular testosterone (ITT) concentration are closely related to spermatogenesis [12, 13]. Testosterone and estradiol are two gonadal hormones produced by the HPG axis. Testosterone is essential to maintain spermatogenesis and male fertility. Spermatogenesis depends on testosterone stimulation. In the absence of testosterone stimulation, spermatogenesis does not proceed beyond the meiosis stage [4, 14]. Estradiol, the predominant form of estrogen, also plays a critical role in male sexual function. Estradiol in men is essential for modulating libido, erectile function, and spermatogenesis. It plays a regulatory role in all stages of spermatogenesis [15]. Previous experimental studies have shown that, in the syndrome of kidney yang deficiency, there are functional disorders of different links and degrees of HPG axis. It is mainly reflected in the decrease of T and cAMP/cGMP levels and the increase of FSH, LH, and E2 levels [16–18]. In this experiment, the levels of cAMP/cGMP and T in the model group were significantly lower than those in the normal group. The levels of LH, FSH, E2, and GnRH in the model group were significantly higher than those in the normal group.

Previous studies have shown that the kidney yang deficiency syndrome model constructed by intragastric administration of adenine shows that the renal tubules of animals are seriously dilated, and the epithelial cells of renal tubules show edema and inflammatory cell infiltration. The diameter of most seminiferous tubules became thinner. The seminiferous epithelium became thinner. The arrangement of spermatogenic cells was disordered, and the number and level of spermatogenic cells decreased. The wall of epididymal tube is thickened, arranged loosely, the interstitial connective tissue is enlarged, the lumen is proliferated and atrophied, and the sperm in the lumen accumulates into a mass [5, 19, 20]. In this study, the kidney volume of the model group increased, and a large number of adenine crystals were found. Some of the crystals were encapsulated by macrophages and multinucleated giant cells, forming granuloma. More renal tubules atrophied and lumen narrowed. The basement membrane of renal tubules was thickened. The renal tubular epithelium became narrow. Necrotic cell fragments were found in a small number of renal tubules. There were more connective tissue hyperplasia and more inflammatory cell infiltration in renal tubulointerstitium. The spermatocytes in seminiferous tubules were disordered. The connective tissue between seminiferous tubules increased. The number of stromal cells decreased significantly. The number of sperm decreased significantly. Seminiferous cells could be seen in some lumens. The epididymal duct epithelial cells were necrotic. The nuclei were pyknotic, stained or fragmented. The lumen was filled with spermatozoa and eosinophilic protein. The interstitial connective tissue was loose, and a small number of inflammatory cells were infiltrated.

The efficacy of HDP includes tonifying kidney, strengthening yang, adding essence, and increasing marrow. It can be used to treat qi and blood deficiency, sallow and emaciated face, nocturnal spermatorrhea, premature ejaculation, impotence, and sore waist and legs. In this experiment, the kidney yang deficiency syndrome (KYDS) was induced by intragastric administration of adenine. Based on the HPG axis, we explored the role of HDP in improving KYDS. From the above experimental results, HDP can improve the general signs of rats with KYDS. Through the detection of organ index, it was found that HDP could improve the atrophy trend of testis, epididymis, and seminal vesicle gland. According to enzyme-linked immunosorbent assays, we determined the serum cytokines of rats and found that HDP can regulate the endocrine disorder of rats. The sperm activity test showed that HDP could improve the sperm activity of rats with KYDS. Histopathological observation showed that HDP could alleviate the pathological damage of testis, epididymis, and seminal vesicle gland to a certain extent. Furthermore, HDP may improve KYDS through other mechanisms, including altered energy metabolism, lipid metabolism, gut microbiota metabolism, and biosynthesis of catecholamine, which is worthy of further study.

## 5. Conclusion

Severe KYDS can lead to abnormal spermatogenesis and infertility. This may be closely related to the hypothalamic-pituitary-gonadal axis. HDP can improve KYDS based on hypothalamic-pituitary-gonadal axis. This study provides a basis for the effectiveness of HDP in the treatment of KYDS.

## Data Availability

The data used to support the findings of this study is available from the corresponding author upon request.

## Abbreviations

HDP: Haima Duobian pill
cAMP: Cyclic adenosine monophosphate
cGMP: Cyclic guanosine monophosphate
LH: Luteinizing hormone
FSH: Follicle-stimulating hormone
T: Testosterone
E2: Estradiol
GnRH: Gonadotropin-releasing hormone
ELISA: Enzyme-linked immunosorbent assays
KYDS: Kidney yang deficiency syndrome
HPG axis: Hypothalamic-pituitary-gonadal axis
TCM: Traditional Chinese Medicine
TCMSP: Traditional Chinese Medicine Systems Pharmacology
NG: Normal group
MG: Model group
HDG: High dose group
MDG: Middle dose group
LDG: Low dose group
PG: Positive group.

## References

[1] Nan Y., Zhou X., Liu Q. (2016). Serum metabolomics strategy for understanding pharmacological effects of Shen Qi pill acting on kidney yang deficiency syndrome. *Journal of Chromatography. B, Analytical Technologies in the Biomedical and Life Sciences*.

[2] Chen R., Wang J., Zhan R., Zhang L., Wang X. (2019). Integrated systems pharmacology, urinary metabonomics, and quantitative real-time PCR analysis to uncover targets and metabolic pathways of the you-gui pill in treating kidney-yang deficiency syndrome. *International Journal of Molecular Sciences*.

[3] Li Z., He Z., Zhang F. (2019). Basic research and clinical application of infertility caused by spermatogenesis dysfunction. *Shanghai Medical Journal*.

[4] Jin J.-M., Yang W.-X. (2014). Molecular regulation of hypothalamus–pituitary–gonads axis in males. *Gene*.

[5] Cui K., Ji B. (2019). Review of modern evaluation index of kidney yang deficiency animal model. *Journal of Gansu University of Chinese Medicine*.

[6] Yao B., Peng R., Wang S. (2000). Preventive and therapeutic effects of a traditional Chinese medicine “Kang Fu Ling” on the sperm injury in rats induced by microwave radiation. *Chinese Journal of Stereology and Image Analysis*.

[7] Unschuld P. U. (2003). *Huang Di Nei Jing Su Wen: Nature, Knowledge, Imagery in an Ancient Chinese Medical Text: With an Appendix: The Doctrine of the Five Periods and Six Qi in the Huang Di Nei Jing Su Wen*.

[8] Gang G., Yang G. (2009). Metabonomic study on urine of rats with hydrocortisone-induced kidney deficiency syndrome. *Academic Journal of Second Military Medical University*.

[9] Lu X., Xiong Z., Li J., Zheng S., Huo T., Li F. (2010). Metabonomic study on ‘Kidney-Yang Deficiency syndrome’and intervention effects of Rhizoma Drynariae extracts in rats using ultra performance liquid chromatography coupled with mass spectrometry. *Journal of Talanta*.

[10] Zou Z., Gong M., Xie Y., Wang S., Liang S. (2018). Urinary metabonomic study of kidney-yang deficiency syndrome induced by hydrocortisone. *Chinese Journal of Experimental Medical Formulae*.

[11] Kaprara A., Huhtaniemi I. T. (2017). The hypothalamus-pituitary-gonad axis: tales of mice and men. *Journal of Metabolism*.

[12] Huhtaniemi I. (2018). Mechanisms in endocrinology: hormonal regulation of spermatogenesis: mutant mice challenging old paradigms. *European Journal of Endocrinology*.

[13] Zirkin B. R. (1998). Spermatogenesis: its regulation by testosterone and FSH. *Seminars in Cell & Developmental Biology*.

[14] Walker W. H. (2010). Non-classical actions of testosterone and spermatogenesis. *Journal of Philosophical Transactions of the Royal Society B: Biological Sciences*.

[15] Schulster M., Bernie A. M., Ramasamy R. (2016). The role of estradiol in male reproductive function. *Asian Journal of Andrology*.

[16] Huang X., Xu Y., Wu J. (2015). Pharmacodynamic study on the therapeutic effect of Wenshen Pill on rats with spermatogenesis disorder due to kidney yang deficiency. *Guizhou Medical Journal*.

[17] Ju C., Li Y., Wang W., Jia T., Ai X. (2020). Analysis on mechanism of adenine-induced kidney-yang deficiency rats treated with different processed products of curculiginis rhizoma. *Chinese Journal of Experimental Traditional Medical Formulae*.

[18] Zheng P., Zhu Y., Ding M. (2019). Establishment of animal model of kidney yang deficiency induced by adenine. *China Journal of Traditional Chinese Medicine and Pharmacy*.

[19] Wang S., Li B., Kan Y. (2015). Duzhong Butiansu Capsules improve adenine-induced reproductive dysfunction in male rats. *National Journal of Andrology*.

[20] Yang X., Wang J., Yang Y., Yan L., Ren L. (2010). Protective effect of Isaria felina mycelia powder against adenine-induced chronic renal injury in mice. *Chinese Remedies & Clinics*.

